# A Rare Case of Primary Ovarian Mucinous Carcinoma with Signet-Ring Cells and Literature Review

**DOI:** 10.5146/tjpath.2026.13669

**Published:** 2026-01-31

**Authors:** Fatma Gundogdu, Alp Usubutun

**Affiliations:** Department of Pathology, Faculty of Medicine, Hacettepe University, Ankara, Türkiye

**Keywords:** Signet-ring cell, Ovary, Krukenberg tumor

## Abstract

The presence of signet-ring cells in the ovary is almost always associated with metastatic mucinous carcinomas known as Krukenberg tumors. Here we report a primary ovarian mucinous carcinoma with signet-ring cells, which is scarcely encountered, and a review of the literature to summarize the clinical and morphological features of these tumors.

The patient was a 26-year-old female who had a large multicystic lesion in the right ovary. Macroscopic examination of the cyst revealed a 30 cm-sized multicystic lesion filled with mucinous material. The capsule was intact, and there was no surface involvement. Microscopically, a multicystic mucinous tumor with a predominantly borderline background and three well-demarcated nodules composed of signet ring cells without desmoplastic stroma were noted in the cyst wall. There was only one invasive focus seen. Immunohistochemically, conventional mucinous areas were diffusely positive for Keratin 7 and Keratin 20, and focally positive for PAX8, while negative for CDX2. Signet ring cells were positive for Keratin 20, CDX2, and Keratin 7, while negative for PAX8. In the systemic examinations, no potential primary site was found. The patient has not received any adjuvant treatment and has been followed for six years without disease, which is the longest follow-up time among previously reported cases.

Signet ring cells can be present in primary ovarian mucinous carcinomas. The distinction from the more frequently seen metastatic carcinomas needs a complete evaluation of clinicopathological findings. Early-stage primary mucinous carcinomas having localized signet-ring cell nodules seem to have favorable prognosis without adjuvant treatment.

## INTRODUCTION

Discussions on ovarian metastatic signet-ring cell tumors, named Krukenberg tumors, go back to the mid-1800s. Notably, the most important diagnostic features of the Krukenberg tumors are the signet-ring cells and accompanying hypercellular stroma. Although the definition of the Krukenberg tumor described in the original paper has changed to date, signet-ring cells are still a significant diagnostic attribute for these tumors. Although it can be seen in various ovarian neoplasms, the existence of signet-ring cells in an ovarian neoplasm is mostly associated with metastatic mucinous carcinomas and particularly metastatic tumors originating from the gastrointestinal system and, less frequently, the breast (1,2). However, signet-ring cells may also be rarely encountered in primary ovarian mucinous carcinomas (3–10). There are 10 primary mucinous tumor with signet-ring cells cases published in the literature to this date (3–10). Distinguishing primary mucinous tumors with signet-ring cells from metastatic ones could be challenging since they have morphological and immunophenotypic similarities. The signet-ring cells can rarely exist in primary ovarian tumors like signet-ring stromal tumors (11). Despite its rarity, non-mucinous tumors containing signet-ring cells also create a challenge for pathologists.

Here, we present a case of primary ovarian mucinous carcinoma containing signet ring cells, with a 6-year clinical follow-up without adjuvant treatment. This is the longest follow-up period among previously reported cases. We mainly discuss the morphological features of the case and review the literature.

## CASE PRESENTATION

**Figure 1:**
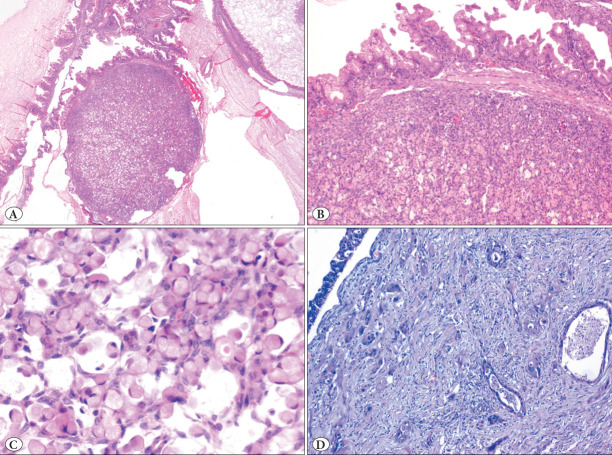
Histopathological features of the neoplasm. **A)** Low power image of the cystic mucinous tumor with 3 mm-sized nodule (H&E 20x). **B)** Higher power magnification showing mucinous borderline tumor morphology in conjunction with the signet-ring cell nodule (H&E 200x) **C)** Signet ring cells with intracytoplasmic mucin (H&E 400x). **D)** Foci of infiltrative invasion (H&E 100x).

**Figure 2:**
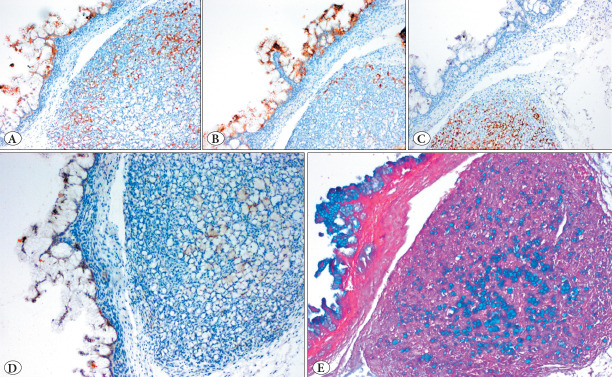
Immunohistochemical features of the neoplasm. **A)** Keratin 20 was diffusely positive in both components (100x). **B)** Keratin 7 was diffusely positive in conventional mucinous areas while focally positive in signet ring cells (100x). **C)** CDX2 was only positive in signet ring cells (100x). **D)** PAX8 was focally positive (100x). **E)** Alcian blue stain highlighted intracytoplasmic mucin (100x).

The patient was a 26-year-old female who was admitted to another hospital with lower abdominal pain. In the abdominal imaging, a sizeable multicystic lesion that reached the epigastrium in the right ovary was seen. A right ovarian cystectomy was performed. Macroscopic examination revealed a 30 cm-sized multicystic lesion filled with mucinous material. The capsule of the lesion was intact, and no surface involvement was seen. The inner surface of the lesion was mostly smooth. A total of 50 blocks were taken from the lesion for microscopic examination. The case was consulted to our center for histopathological confirmation and further management. Microscopically, a mucinous tumor that showed a multicystic appearance with a dominant borderline tumor background was seen (Figure 1). A total of 3 well-delineated nodules sized 0.3 cm, 0.2 cm, and 0.1 cm and composed of signet ring cells organized in nests or single cells separated by delicate fibrous septa without notable stroma were observed in the cyst wall (Figure 1). Signet ring cells were recognized by their intracytoplasmic mucin and eccentrically displaced nuclei (Figure 1). Signet-ring cells were only found in these three well-circumscribed foci. There was only one focus of malignant tumor with infiltrative/destructive invasion and there was no signet-ring cell in this invasive focus (Figure 1). No teratomatous elements, multi-nodularity, lymphovascular invasion (LVI), or surface involvement was present. Immunohistochemically, there was diffuse positivity for Keratin 20 in both the conventional mucinous tumor and signet ring cells (Figure 2). Keratin 7 was diffusely expressed in conventional mucinous areas while focally expressed in signet ring cells (Figure 2). CDX2 was positive in signet ring cells, in contrast to negative staining in other areas (Figure 2). PAX8 was focally positive in borderline mucinous areas while negative in signet ring cells (Figure 2). No loss of SMAD4 expression was observed. Alcian blue highlighted the intracytoplasmic mucin of signet ring cells (Figure 2).

Based on these findings, the diagnosis was made as mucinous adenocarcinoma with signet ring cells. Due to the existence of signet ring cells, gastrointestinal system examination was recommended to exclude possible metastatic tumors. No pathology was detected in endoscopic and colonoscopic examinations. Then, the patient underwent fertility-sparing surgery (right salphingooopherectomy + left ovarian cystectomy + lymph node dissection + omentectomy). Examination of the specimens showed no tumor involvement in the left ovary, lymph nodes, or omentum. No further treatment was planned.

The patient was followed for three years without treatment and any recurrence or metastasis and due to a radiologically suspected left ovarian cystic lesion a complementary surgery (hysterectomy + left salphingooopherectomy) was done. However, no significant pathological finding was found in the macroscopic and microscopic examination of the specimen. The patient has been monitored without recurrence or metastasis for three years after the complementary surgery and for a total of six years since the diagnosis.

## DISCUSSION

In the ovary, signet-ring cells primarily exist in metastatic mucinous carcinomas, the so-called Krukenberg tumor. Possible primary sites for the Krukenberg tumor include the gastrointestinal tract (most likely), appendix, pancreaticobiliary system, urinary bladder, or lobular breast carcinoma (1,2). The existence of signet ring cells is rarely reported in primary ovarian mucinous tumors (3–10). Distinguishing primary and metastatic mucinous tumors may be a real challenge due to shared histopathological and immunophenotypic features. While bilaterality, ovarian surface involvement, multinodularity, and LVI are features that suggest metastasis; low tumor stage, mucinous adenoma/adenofibroma background, and presence of endometriosis favor primary tumor (9). Tumor stroma could also be a clue since it is more likely to be hypercellular or sometimes edematous in Krukenberg tumors (2). The presented patient had FIGO stage IA unilateral tumor without surface involvement or LVI in a mostly mucinous borderline tumor background and showed focal PAX8 positivity. There was a single destructive invasive focus. Signet ring cells appeared as three well-circumscribed small nodules in the cyst wall with no prominent stromal hypercellularity, edema, or desmoplasia. Further systemic workup revealed no possible primary site; the patient had been followed for 6 years without any disease. Based on these features, we are confident to call this case a primary ovarian mucinous carcinoma with signet ring cells.

As far as we know, there are 10 reported primary ovarian mucinous carcinoma with signet ring cells cases to this date (3–10). There are also a few primary ovarian mucinous carcinomas containing signet ring cells developing from mature cystic teratoma cases reported (12,13). But neither the previously reported cases nor our case had any teratomatous component. Important clinicopathological characteristics of the reported cases are outlined in Table 1. These tumors were seen in a wide range of ages between 24 and 78, averaging 47.9. Tumor sizes varied between 9 to 30 cm, averaging 20.1 cm. In most cases (8/11), signet ring cells appeared as invasive foci within a hypo/moderately cellular stroma. But the other 3 cases, including ours, showed well-demarcated signet ring cell nodules varying in sizes up to 5 cm (3,4). In 3 of the 11 cases, the predominant component was signet ring cells (5,7,8) while, in others, signet ring cell focuses were noted focally with sizes that varied between 0.1 cm and 5 cm (3,4,6,9,10). None of the cases had hypercellular/desmoplastic stroma, which would be expected to be seen in the Krukenberg tumor. Based on summarized features in Table 1, we can say that primary ovarian mucinous carcinomas containing signet ring cells tend to be unilateral, low-stage tumors with mostly mucinous adenoma/borderline tumor background or endometriosis, without ovarian surface involvement, LVI, or multinodularity. Three of the patients received adjuvant paclitaxel/carboplatinum chemotherapy (3–5). Only one of the patients showed recurrent disease after 2 years of diagnosis (3) and one patient died due to pulmonary complications were after treatment (5). The relatively short follow-up times of the cases have varied between 8 months and 3 years. Our patient has the longest follow-up among the cases, with 6 years of disease-free survival. The prognosis of these tumors is unclear since there are few cases with relatively short follow-up times. However, based on the above mentioned follow-up data, we can suggest that these tumors can be followed up without further treatment after the surgery, and adjuvant chemotherapy can be used in higher-stage tumors.

**Table 1:** The summary of clinicopathological features of previously reported cases and current case

**Cases**	**Age**	**Laterality**	**Whole tumor size (cm)**	**Signet ring cell component size**	**Capsule**	**Surface involvement**	**Extracellular mucin**	**LVI**	**Multinodularity**	**Accompanying pathology**	**FIGO stage**	**Surgery**	**Adjuvant treatment**	**Follow-up**	**Survival**
Ong and Ostör (3)	60	right	15	5 cm	intact	yes	no	no	no	no	IIIB	H+BSO+A+ O+PPLND	yes**	3 years	Recurrence in 2 years
McCluggage WG and Young (9)															
Case 1	27	right	9	1 cm (10% of the tumor)	intact	no	no	no	no	mucinous cystadenoma	IA	USO	no	3 years	W/A
Case 2	55	left	9	Small focal areas (10% of the tumor)	intact	no	no	no	no	adenofibromatous background	IA	H+BSO +A+O	no	NA	NA
Case 3	60	left	27	N/A	intact	no	no	no	no	mucinous cystadenoma/ borderline tumor	IA	H+BSO +A+O	no	8 months	W/A
El-Safadi et al. (5)	24	right*	25	Most of the tumor	N/A	yes	yes	yes	-	no	IIIC	USO+O+ A	yes	2 years	Exitus ***
Jaya Ganesh et al. (10)	38	right	20	3 cm	focal nodularity	no	yes	no	no	no	IC	H+BSO + O	N/A	LOF	NA
Kim et al. (8)	54	right	20.5	Most of the tumor	intact	no	no	no	no	mucinous cystadenoma/ borderline tumor	IA	H+BSO + A + O	no	1 year	W/A
Jiao et al. (6)	46	right	18	1 cm	focal disruptions	no	yes	no	no	endometriotic cyst	IC	H+BSO +A + O+ PPLND	no	13 months	W/A
Khadang and Omeroglu (7)	78	right	24	Most of the tumor	intact	no	yes	no	no	benign/malignant Brenner tumor	IA	H+BSO +O	no	1 year	W/A
Pongsuvareeyakul et al. (4)	59	left	24	3 cm and 0.5 cm	ruptured	no	no	no	no	focal neuroendocrine differentiation	IC2	BSO+O	yes	11 months	W/A
Present case	26	right	30	0.3 cm, 0.2 cm and 0,1 cm	intact	no	no	no	no	mucinous cystadenoma/borderline tumor	IA	USO+O + A+PPLND	no	6 years	W/A

**W/A:** Well and Alive, **NA:** Not Available, **LOF:** Loss of Follow-up, **H:** Hysterectomy, **USO:** Unilateral Salphingooopherectomy, **BSO: **Bilateral Salphingooopherectomy, **A:** Appendectomy, **O: **Omentectomy, **PPLND:** Pelvic Paraaortic Lymph Node Dissection

*Left ovary that had a mucinous borderline tumor removed one year ago

** Recurrent disease treated with Paclitaxel and Carboplatin chemotherapy

*** Due to pulmonary complications

The benefit of immunohistochemistry is limited in distinguishing primary and secondary ovarian mucinous carcinomas with signet ring cells due to immunophenotypic similarities. Primary carcinomas are generally diffusely positive for keratin 7, while there is variable positivity for keratin 20, CEA, and CDX2. SMAD4 could be helpful since its loss would support a metastatic tumor. In our case, neoplastic cells expressed the intestinal markers in varying proportions but also showed focal PAX8 expression in mucinous cells, which supported the primary origin.

Besides malignant tumors, signet-ring cells may draw attention in some benign ovarian tumors as well, like signet-ring stromal tumors, a benign sex cord-stromal tumor that is characterized by signet-ring cells in a fibromatous stroma. The distinction from primary or metastatic signet-ring cell carcinomas can be made based on the absence of EMA immunopositivity and intracytoplasmic mucin in signet-ring stromal tumors (11).

In conclusion, signet ring cells might rarely be appreciated in primary ovarian mucinous carcinomas. The distinction from more frequently seen metastatic signet ring cell carcinomas needs a complete evaluation of clinical and pathological findings. Early-stage, low-grade primary mucinous carcinomas having localized signet-ring cell nodules without invasion seem to have favorable prognoses without adjuvant treatment after surgery.

## Conflict of Interest

The authors have no conflict of interest.

## References

[ref-1] Kiyokawa Takako, Young Robert H., Scully Robert E. (2006). Krukenberg tumors of the ovary: a clinicopathologic analysis of 120 cases with emphasis on their variable pathologic manifestations. Am J Surg Pathol.

[ref-2] Young Robert H. (2006). From krukenberg to today: the ever present problems posed by metastatic tumors in the ovary: part I. Historical perspective, general principles, mucinous tumors including the krukenberg tumor. Adv Anat Pathol.

[ref-3] Ong Nicole C. S., Ostör Andrew G. (2002). Borderline mucinous tumour with mural nodule of signet ring adenocarcinoma. Aust N Z J Obstet Gynaecol.

[ref-4] Pongsuvareeyakul Tip, Charoenkwan Kittipat, Suprasert Prapaporn, Khunamornpong Surapan (2020). Primary signet ring cell carcinoma with neuroendocrine differentiation arising in mucinous borderline tumor of the ovary. Gynecol Oncol Rep.

[ref-5] El-Safadi Samer, Stahl Ulrich, Tinneberg Hans Rudolf, Hackethal Andreas, Muenstedt Karsten (2010). Primary signet ring cell mucinous ovarian carcinoma: a case report and literature review. Case Rep Oncol.

[ref-6] Jiao Yurong, Lu Bingjian (2019). Poorly differentiated mucinous carcinoma with signet ring cells in an ovarian endometriotic cyst: a case report. Diagn Pathol.

[ref-7] Khadang Baharak, Omeroglu Atilla (2020). Ovarian Mixed Malignant Brenner-Mucinous Tumor with Signet Ring Cells. Case Rep Pathol.

[ref-8] Kim Ji Hye, Cha Hee Jeong, Kim Kyu-Rae, Kim Kyungbin (2018). Primary ovarian signet ring cell carcinoma: A rare case report. Mol Clin Oncol.

[ref-9] McCluggage W. Glenn, Young Robert H. (2008). Primary ovarian mucinous tumors with signet ring cells: report of 3 cases with discussion of so-called primary Krukenberg tumor. Am J Surg Pathol.

[ref-10] P Jaya Ganesh, R Vimal Chander, P Kanchana M., Narasimhan Lakshmi (2014). Primary ovarian mucinous carcinoma with signet ring cells - report of a rare case. J Clin Diagn Res.

[ref-11] Matsumoto Manabu, Hayashi Yoshihiro, Ohtsuki Yuji, Ikegami Nobuo, Toi Makoto, Iguchi Mitsuko, Hiroi Makoto (2008). Signet-ring stromal tumor of the ovary: an immunohistochemical and ultrastructural study with a review of the literature. Med Mol Morphol.

[ref-12] Zheng Hong-fang, Jiang Bao-yu, Shen Dan-hua (2005). [Signet ring cell carcinoma arising from mature cystic teratoma of the ovary]. Zhonghua Bing Li Xue Za Zhi.

[ref-13] Vang Russell, Gown Allen M., Zhao Chengquan, Barry Todd S., Isacson Christina, Richardson Mary S., Ronnett Brigitte M. (2007). Ovarian mucinous tumors associated with mature cystic teratomas: morphologic and immunohistochemical analysis identifies a subset of potential teratomatous origin that shares features of lower gastrointestinal tract mucinous tumors more commonly encountered as secondary tumors in the ovary. Am J Surg Pathol.

